# Sex differences in ex vivo cytokine production and their association with outcome following ischemic stroke

**DOI:** 10.3389/fimmu.2026.1856274

**Published:** 2026-07-28

**Authors:** Marcin Piechota, Michal Korostynski, Kazimierz Weglarczyk, Maciej Siedlar, Tomasz Dziedzic

**Affiliations:** 1 Laboratory of Pharmacogenomics, Maj Institute of Pharmacology, Polish Academy of Sciences, Krakow, Poland; 2 Department of Clinical Immunology, Institute of Pediatrics, Jagiellonian University Medical College, Krakow, Poland; 3 Department of Neurology, Jagiellonian University Medical College, Krakow, Poland

**Keywords:** angiotensin receptor blockers, cytokines, gene expression, outcome, sex, stroke

## Abstract

**Background:**

Animal studies suggest that post-stroke inflammatory responses are sexually dimorphic and may influence recovery. We examined whether men and women with acute ischemic stroke differ in ex vivo cytokine synthesis and whether such differences relate to functional outcome.

**Methods:**

We included 279 patients with acute ischemic stroke (mean age: 68.5 ± 12.8; 41.2% women). Blood samples collected on day 3 after stroke were stimulated with lipopolysaccharide (LPS). Ex vivo production of TNFα, IP-10, IL-1β, IL-6, IL-8, IL-10, and IL-12p70 was measured using enzyme-linked immunoassay or cytometric methods, and expression of LPS-induced cytokine and chemokine genes was assessed by RNA sequencing.

**Results:**

Greater stroke severity, lower monocyte counts, and the use of angiotensin receptor blockers were associated with decreased production of pro-inflammatory cytokines. Compared with females, males showed higher ex vivo synthesis of TNFα (mean: 2814.2 vs 2302.7 pg/mL, P = 0.003), IL-1β (1851.9 vs 1461.3 pg/mL, P = 0.002), IL-6 (14345.3 vs 11846.5 pg/mL, P = 0.004), IL-12 (7.1 vs 4.2 pg/mL, P = 0.002), and IP-10 (557.9 vs 435.1 pg/mL, P = 0.038), with correspondingly higher gene expression levels of TNFα, IL-1β, IL-6, and IL-12. In multivariable quantile regression models adjusted for cytokine-specific covariates, male sex remained significantly associated with higher levels of TNFα, IL-1β, and IL-6. Despite this sex-related difference in inflammatory response, three-month functional outcomes were similar in men and women.

**Conclusion:**

Males exhibited greater ex vivo pro-inflammatory cytokine production after ischemic stroke, but this sexual dimorphism did not translate into differences in functional recovery.

## 1 Introduction

The immune response plays an important role in stroke pathophysiology, shapes outcomes and could be the potential therapeutic target (1). There is accumulating evidence that both central and peripheral post-stroke inflammatory reactions are sexually dimorphic and determine differences in infarct size and post-stroke recovery between males and females (2, 3). In experimental models of ischemic stroke, males had a greater number of activated macrophages/microglia, CD8+ and regulatory T cells in the brain than females (4, 5). In turn, females had more anti-inflammatory macrophages/microglia and regulatory B cells in the ischemic hemisphere (6). After middle cerebral artery occlusion, the percentage of CD4+ T lymphocytes was significantly higher in the spleens of males whereas females had a greater percentage of CD8+ T lymphocytes and interleukin (IL)-10-secreting suppressor T cells than males (5).

Some animal studies reported that the response to immune-based therapy after stroke could be sex-dependent. Leukemia inhibitory factor (LIF) exerted stronger anti-inflammatory effects on the post-stroke splenic response in aged female rats as compared to males (7). Splenectomy reduced infarct size and neuroinflammation in male but not female mice (8). Minocycline, a prototypic anti-inflammatory agent, diminished neuroinflammation and ischemic damage only in males (9).

Sex-specific differences in the immune response following stroke have also been reported in some clinical studies. A significantly stronger immune response in the protein-coding peripheral blood transcriptome was observed in women with ischemic stroke compared to men. The innate immune response pathways were overrepresented in females including cytokines (e.g. IL-1, IL-6, IL-12, IL-18), Toll-like receptor (TLR), transforming growth factor-beta and IL-17 signaling genes (10–12). While these transcriptomic studies provide a valuable snapshot of systemic gene activity, they do not capture the functional capacity of immune cells to elicit an inflammatory response.

Whole blood stimulation assays are powerful tools to investigate the ability of immune cells to respond to different stimuli (13). In these assays, blood is collected into anticoagulant-containing tubes, divided into aliquots, and incubated with specific stimulants or antigens. Subsequently, either the supernatant or the remaining cellular fraction is analyzed to characterize the induced immune response. In many studies, cytokine concentrations in the supernatant serve as the primary measure of immune activation. These assays provide valuable insight into functional alterations of the immune system that may not be detectable under resting conditions. Whereas circulating cytokine levels reflect ongoing inflammatory processes *in vivo*, cytokine production following ex vivo whole blood stimulation represents the responsiveness and functional potential of immune cells when challenged with specific triggers. Therefore, these two measures carry different biological meanings and may relate to stroke outcomes in distinct ways.

TLR-4 plays a central role in the innate immune response. The stimulation of this receptor with its potential ligand, lipopolysaccharide (LPS), induces the release of pro-inflammatory cytokines, providing a direct measure of an individual’s inflammatory potential (14). Using whole blood stimulation with LPS, we previously demonstrated that a reduced ex vivo synthesis of tumor necrosis factor alpha (TNFα) and other pro-inflammatory cytokines in acute ischemic stroke patients is associated with an increased risk of poor functional outcomes and adverse events like pneumonia, delirium and fatigue (15–19).

A better understanding of sex differences in immune response to stroke might be important for more tailored therapeutic strategies. Our study aimed to determine (1) if there is any difference in ex vivo cytokine synthesis after LPS stimulation between men and women with acute ischemic stroke and (2) whether these differences are associated with sex-dependent functional outcome.

## 2 Methods

### 2.1 Patient selection and clinical assessment

The patient cohort for this secondary analysis was derived from a prospective study that explored the relationship between ex vivo cytokine synthesis and outcomes after ischemic stroke (17, 20). That study was conducted in the Department of Neurology, University Hospital, Krakow, Poland, between October 2016 and February 2019. We screened consecutive patients admitted to our department due to acute stroke. The inclusion criteria were: (1) ischemic stroke; (2) time from the onset of stroke symptoms to admission <24 h; (3) pre-stroke modified Rankin Scale (mRS) score of 0–2 (independent of daily activities); (4) National Institute of Health Stroke Scale (NIHSS) score on admission >3; and (5) informed patient consent. The exclusion criteria were: (1) chronic inflammatory, autoimmune, or cancerous diseases; (2) the use of steroids or immunomodulatory drugs before stroke or in the acute phase of stroke; (3) pre-stroke diagnosis of depression and/or the use of anti-depressive medication. An NIHSS score >3, predefined in the parent study, was selected to exclude only the mildest strokes, which are associated with ceiling effects in functional outcomes and a blunted inflammatory response, while preserving a broad spectrum of moderate-to-severe strokes.

The Bioethics Committee of Jagiellonian University approved the study protocol (decision number: 122.6120.249.2016) and each participant gave informed consent.

We used the NIHSS to assess neurological deficit on admission and mRS to assess functional outcome 3 months after stroke. Poor outcomes were defined as a mRS score of 3–6. We determined stroke etiology according to the Trial of Org 10172 in Acute Stroke Treatment (TOAST) classification.

### 2.2 Laboratory methods

The methodology of whole blood stimulation and RNA sequencing was previously described (17, 20, 21). Briefly, venous blood was collected in heparinized tubes (Sarstedt, Nuembrecht, Germany) at day 3 after stroke. Given the highly dynamic nature of the early inflammatory response following stroke, day 3 was selected to minimize variability in cytokine synthesis related to the interval between symptom onset and blood stimulation. To avoid diurnal variation, blood was obtained between 7:00 AM and 7:30 AM. Within 30 min of collection, the whole blood was diluted by 1:5 in sterile RPMI 1640 medium supplemented with L-glutamine (Sigma–Aldrich, St. Louis, MO) and stimulated in sterile glass tubes (Lonza, Walkersville, MD) at 37 °C in 5% CO_2_ with LPS (10 ng/mL, *E. coli* 0111:B4, Sigma–Aldrich, St. Louis, MO). Blood stimulation was performed for 4 h for TNFα and interferon gamma-induced protein 10 (IP-10), and 24 h for IL-1β, IL-6, IL-8, IL-10, and IL-12p70. The supernatants were removed and stored at −80 °C until further analysis. TNFα and IP-10 concentrations were measured using a commercially available enzyme-linked imunoassay (ELISA) kit (R&D Systems, Minneapolis, MN) according to the manufacturer’s instructions. The levels of IL-1β, IL-6, IL-8, IL-10, and IL-12p70 were determined by a cytometric bead array (Human Inflammatory Kit, BD Biosciences, San Diego, CA).

After whole blood stimulation with LPS for 4 h, the PAXgene Blood RNA Tube reagent (PreAnalytiX, GmbH, Switzerland) was added and obtained blood lysates were transferred to PAXgene tubes and stored at -80 °C for subsequent RNA analysis. Total RNA was isolated from blood using the PAXgene Blood RNA Kit (Qiagen, Valencia, CA) following the manufacturer’s protocol. RNA concentrations were measured using the NanoDrop ND-1000 Spectrophotometer (NanoDrop Technologies, Montchanin, DE) and RNA quality was determined by chip-based capillary electrophoresis utilizing the Agilent RNA 6000 Pico Kit (Agilent, Santa Clara, CA) and Agilent Bioanalyzer 2100 (Agilent, Palo Alto, CA). Samples with poor quality RNA (RNA integrity number < 7) were excluded from gene expression profiling. The mRNA libraries were generated with NEBNext Ultra II Directional RNA Library Prep Kit for Illumina (New England Biolabs, Ipswich, MA). The polyA transcriptome libraries were sequenced on NovaSeq 6000 (Illumina) sequencer with the following parameters: PE 150 (paired ends) and 40 M clean reads, which yielded more than 12 Gb of raw data per each sample. The quality of next-generation sequencing data was verified using FastQC v. 0.11.8. The RNA-sequencing reads were aligned to GRCh38.p13 using Hisat2 v. 2.0.5. The transcript Fragments Per Kilobase of transcript, per Million mapped reads were quantified using Cufflinks v. 2.2.1 and General Transfer Format from the Ensembl gene database.

### 2.3 Statistical analysis

We used the Student t-test and the χ^2^ test to compare baseline characteristics and LPS-induced cytokines between males and females.

To gain a more comprehensive understanding of the association between sex and cytokine production, we performed quantile regression analyses with cytokine levels as dependent continuous variables. This approach does not require assumptions regarding the distribution residuals and is less sensitive to outliers than conventional linear regression (22). First, we conducted univariable analyses to identify clinical variables (age, sex, vascular risk factors, NIHSS score, reperfusion therapy and pre-stroke medication) and laboratory variables (monocyte and lymphocyte counts) associated with ex vivo cytokine synthesis. These analyses were performed separately for each cytokine. Subsequently, we examined the association between sex and cytokine levels using multivariable quantile regression models. For each cytokine, the models were adjusted for a cytokine-specific set of potential confounders, including variables that showed an association in univariable analyses at a significance level of P < 0.10.

We subsequently compared the expression levels of LPS-induced cytokine and chemokine genes between males and females. Cytokines and chemokines included in this analysis were selected based on previous studies (23–25). We used Fragments Per Kilobase of transcript per Million mapped reads (FPKM) values as the measure of transcript abundance levels. We assessed differences in gene expression using multivariable analysis of covariance (ANCOVA), with log2-transformed FPKM values as the depend variable, sex as the independent variable, and age, NIHSS score and monocyte count included as pre-specified covariates. To account for multiple testing across numerous transcripts, we applied the Benjamini-Hochberg procedure to control the False Discovery Rate (FDR). Statistical significance was defined as an FDR-adjusted P value below 0.10.

We performed univariable and multivariable logistic regression analyses to assess the associations of cytokine levels and sex with poor functional outcome at 3 months. Multivariable models were adjusted for covariates associated with the outcome in univariable analyses at a significance level of P < 0.10. Optimal cytokine cut-off values for predicting functional outcomes were determined using receiver operating characteristic (ROC) curve analysis and the Youden index.

The calculations were performed using STATISTICA (version 13.3, TIBCO, Poland) and R (version 4.1.1) software. Data are presented as means with standard deviations (SD).

## 3 Results

We included 279 ischemic stroke patients (mean age: 68.5 ± 12.8; mean NIHSS score on admission: 11.3 ± 6.6; 41.2% women).

In the whole group of our patients, women were older and more often suffered from hypertension and atrial fibrillation compared with men. In turn, men were more frequently smokers and suffered from ischemic heart disease (**Table 1**).

**Table 1:** Baseline characteristics of patients and outcomes.

	Stroke patients		
	Female (N = 115)	Male (N = 164)	P value
Age, mean (SD)	73.6 (12.1)	64.9 (12.1)	<0.001
NIHSS score on admission, mean (SD)	11.4 (6.7)	11.3 (6.6)	0.938
Vascular factors			
Hypertension, n (%)	97 (84.3)	122 (74.4)	0.046
Ischemic heart disease, n (%)	11 (9.6)	31 (18.9)	0.032
Atrial fibrillation, n (%)	43 (37.4)	39 (23.8)	0.014
Diabetes mellitus, n (%)	38 (33.0)	42 (25.6)	0.176
Previous stroke, n (%)	10 (8.7)	24 (14.6)	0.135
Smoking, n (%)	19 (16.5)	52 (31.7)	0.004
*Stroke etiology*			0.090
Large-artery atherosclerosis, n (%)	27 (23.5)	51 (31.1)	
Cardioembolic stroke, n (%)	44 (38.3)	39 (23.8)	
Small-vessel disease, n (%)	4 (3.5)	10 (6.1)	
Undetermined cause, n (%)	38 (33.0)	58 (35.3)	
Other determined cause, n (%)	2 (1.7)	6 (3.7)	
In-hospital therapy			
Reperfusion therapy, n (%)	66 (57.4)	109 (66.5)	0.123
Antibiotics, n (%)	39 (33.9)	49 (29.9)	0.475
Pre-stroke medication			
Acetylsalicic acid, n (%)	29/107 (27.1)	41/145 (28.3)	0.837
Statins, n (%)	26/107 (24.3)	40/154 (26.0)	0.759
β-blockers, n (%)	54/105 (51.4)	46/138 (33.3)	0.005
α-blockers, n (%)	3/105 (2.9)	19/138 (13.8)	0.003
Diuretics, n (%)	42/106 (39.6)	32/139 (23.0)	0.005
ACE-Is, n (%)	32/105 (30.5)	45/138 (32.6)	0.723
ARBs, n (%)	20/105 (19.0)	8/138 (5.8)	0.001
Insulin, n (%)	11/114 (9.6)	12/156 (7.9)	0.569
Oral hypoglycemic drugs, n (%)	27/114 (23.7)	24/157 (15.3)	0.081
*In hospital pneumonia, n (%)*	7 (6.1)	13 (7.9)	0.726
Outcomes			
90-day poor functional outcome, n (%)	57/111 (51.3)	75/161 (46.6)	0.439
90-day mortality, n (%)	16/111 (14.4)	22/161 (13.7)	0.861

ACE-Is, angiotensin converting enzyme inhibitors; ARBs, angiotensin receptors blockers; NIHSS, National Institute of Health Stroke Scale.

Males demonstrated a higher synthesis of TNFα, IL-1β, IL-6, IL-12 and IP-10 (**Table 2**).

**Table 2:** Cytokine levels and cell counts.

	Stroke patients		
	Female (N = 115)	Male (N = 164)	P value
Ex vivo cytokines (pg/mL)			
TNFα	2302.7 (1469.1)	2814.2 (1384.5)	0.003
IP-10	435.1 (400.6)	557.9 (535.4)	0.038
IL-1β	1461.3 (1003.6)	1851.9 (1014.8)	0.002
IL-6	11846.5 (7160.8)	14345.3 (7017.7)	0.004
IL-8	2449.8 (2829.6)	2365.3 (1861.9)	0.764
IL-10	62.0 (42.6)	62.2 (42.4)	0.972
IL-12	4.2 (4.4)	7.1 (9.0)	0.002
White blood cells count, ×10^3^/µL	9.1 (3.5)	9.1 (3.0)	0.943
Monocyte, ×10^3^/µL	0.8 (0.3)	0.9 (0.3)	0.063
Lymphocyte, ×10^3^/µL	1.8 (0.8)	2.0 (1.2)	0.118

Values are shown as means with standard deviation.

To further investigate the association between sex and cytokine production, we performed quantile regression analyses. In univariable analysis, male sex was related to the increased production of TNFα (β: 587.3, 95%: 366.1 – 819.2, P<0.01), IL-1β (β: 511.5, 95%: 350.5 – 704.6, P<0.01), IL-6 (β: 3179.3, 95%: 1163.8 – 4573.3, P<0.01) and IP-10 (β: 112.7, 95%: 24.0 – 180.8, P = 0.02) (**Supplementary Table 1**).

In multivariable quantile regression models adjusted for cytokine-specific covariates, male sex remained significantly associated with higher levels of TNFα (β: 407.28, 95%CI: 113.05 - 668.47, P = 0.02; adjusted for NIHSS score, monocyte count, lymphocyte count, and angiotensin receptor blockers [ARBs] use), IL-1β (β: 310.07, 95% CI: 187.01 - 514.00, P = 0.01; adjusted for monocyte count, hypertension, reperfusion therapy, and ARBs use), and IL-6 (β: 1974.55, 95% CI: 5.58 - 3064.27, P = 0.04; adjusted for monocyte count, ischemic heart disease, and ARBs use) (**Supplementary Table 2**).

For IL-10 synthesis, there was a significant interaction between sex and monocyte count (β: -50.42, 95% CI: −78.22 to −18.36, P = 0.04). Among females (reference group), higher monocyte count was associated with an increased median IL-10 production (β: 75.18, 95% CI: 28.22 - 99.35, P<0.01). In males, this association was significantly attenuated, as the effect of monocyte count was 50.42 units lower than that observed in females, corresponding to an estimated effect of 24.76 units (75.18 − 50.42) (**Supplementary Table 2**).

Among the other independent variables, a higher NIHSS score was associated with reduced release of TNFα, IL-8, IL-10, IL-12, and IP-10, whereas a higher monocyte count was associated with increased release of TNFα, IL-1β, IL-6, IL-8, IL-10, and IL-12 in univariable analyses. In addition, the use of ARBs was associated with decreased release of TNFα, IL-1β, IL-6, IL-12, and IP-10 in univariable analyses. In multivariable analyses, monocyte count remained independently associated with the release of TNFα, IL-1β, IL-6, IL-8, IL-10, and IL-12, NIHSS score remained associated with TNFα and IP-10 release, and the ARBs use remained associated with IL-1β and IL-12 release (**Supplementary Tables 1**, **2**).

To validate these findings at the transcriptional level, we compared mRNA expression for selected LPS-induced cytokines and chemokines between males and females. RNA sequencing was performed in whole blood samples stimulated with LPS. The procedure was performed for the first 200 participants. After exclusion of samples with poor quality RNA (N = 9), we included 74 women and 117 men. Women were older than men (mean: 71.3 ± 13.1 vs 64.3 ± 11.7, P<0.001), but groups did not differ in NIHSS scores (mean: 10.0 ± 6.0 vs 9.7 ± 5.8, P = 0.712) and monocyte counts (mean: 0.8 ± 0.3×10^3^/µL vs 0.9 ± 0.3×10^3^/µL, P = 0.097). After adjusting for age, stroke severity and monocyte count, gene expression of TNFα, IL-1β, IL-6 and IL-12 was up-regulated in males as compared to females (**Table 3**).

**Table 3:** Cytokine and chemokine gene expression in males and females.

Gene/transcript	Female mean FPKM	Male mean FPKM	t test t (df = 189)	t test P value/FDR	ANCOVA* F (df = 1)	ANCOVA* P value/FDR
IL6 ENST00000258743	679.92	893.63	-3.605	0.000399 0.0108	14.068	0.000236 0.00637
IL6 ENST00000426291	1266.48	1550.93	-2.869	0.00458 0.0309	9.47	0.00241 0.0325
IL12B ENST00000231228	120.77	164.44	-2.936	0.00374 0.0337	9.463	0.00242 0.0217
IL12A ENST00000480088	1.57	2.03	-3.011	0.00296 0.04	8.949	0.00316 0.0213
TNF ENST00000449264	703.09	873.83	-2.801	0.00563 0.0304	8.516	0.00396 0.0214
IL1B ENST00000263341	3015.27	3346.33	-2.481	0.014 0.0629	7.539	0.00664 0.0299
IL6 ENST00000404625	4.93	6.25	-2.348	0.0199 0.0768	6.602	0.011 0.0424
CCL8 ENST00000394620	166.51	198.86	-1.547	0.124 0.257	5.518	0.0199 0.0671
CXCL10 ENST00000306602	150.03	194.81	-1.918	0.0566 0.17	5.496	0.0201 0.0604
IL6 ENST00000401630	471.98	608.14	-2.147	0.033 0.112	5.153	0.0244 0.0658
IL6 ENST00000464710	2.51	3.07	-1.725	0.0861 0.232	3.426	0.0658 0.161
IL6 ENST00000407492	7.63	10.16	-1.643	0.102 0.25	3.278	0.0719 0.162
IL6 ENST00000485300	34.35	40.06	-1.616	0.108 0.242	2.711	0.101 0.211
IL1B ENST00000416750	1.93	2.16	-1.336	0.183 0.33	1.749	0.188 0.362
CCL2 ENST00000580907	22.95	20.49	1.406	0.161 0.311	1.696	0.194 0.35
CCL2 ENST00000582017	7.8	7.06	1.218	0.225 0.379	1.169	0.281 0.474
CXCL8 ENST00000307407	1218.14	1201.01	0.131	0.896 0.93	0.414	0.521 0.827
IL1B ENST00000487639	26.65	27.8	-0.523	0.602 0.812	0.363	0.547 0.821
IL10 ENST00000471071	2.17	2.31	-0.605	0.546 0.776	0.328	0.568 0.807
CXCL8 ENST00000401931	102.23	92.59	0.831	0.407 0.646	0.249	0.618 0.835
CXCL8 ENST00000483500	33.36	30.36	0.767	0.444 0.666	0.224	0.636 0.818
IL1B ENST00000491056	16.76	16.2	0.441	0.66 0.81	0.222	0.638 0.783
IL10 ENST00000423557	4.15	3.97	0.458	0.647 0.832	0.164	0.686 0.805
CCL2 ENST00000225831	1850.22	1819.63	0.207	0.836 0.903	0.125	0.724 0.814
IL1B ENST00000496280	31.99	32.9	-0.309	0.758 0.853	0.105	0.747 0.806
IL1B ENST00000477398	18.43	17.87	0.368	0.713 0.837	0.074	0.786 0.817
IL6 ENST00000401651	5.36	5.44	-0.106	0.916 0.916	0.017	0.898 0.898

*model adjusted for age, stroke severity and monocyte count.

Three-month functional outcome did not differ between men and women (**Table 1**). In univariable logistic regression analysis, lower levels of TNFα, IP-10, IL-1β, and IL-12, as well as higher levels of IL-8 and IL-10, were associated with poor functional outcome at 3 months after stroke (**Supplementary Table 3**). Other predictors of poor outcome included older age (OR: 1.04, 95% CI: 1.02 – 1.07, P<0.01), higher NIHSS score (OR: 1.13, 95% CI: 1.08 – 1.17, P<0.01), and higher monocyte count (OR: 6.92, 95% CI: 2.91 – 16.46, P<0.01). Sex was not associated with function outcome in univariable analysis (OR: 1.21, 95%CI: 0.74 – 1.97, P = 0.44). After adjustment for age, NIHSS score, and monocyte count, lower levels of TNFα, IP-10, IL-1β, IL-12, as well as higher levels of IL-10, remained significantly associated with poor outcome. These results remained essentially unchanged after additional adjustment for sex. The association between sex and functional outcome was non-significant across all multivariable models (P >0.40). Furthermore, no significant interaction was observed between sex and cytokine levels with respect to functional outcome.

## 4 Discussion

Our study demonstrated sexual dimorphism in the innate immune response following ischemic stroke, with males exhibiting higher ex vivo synthesis and gene expression of pro-inflammatory cytokines compared with females. Sex-dependent differences in TNFα, IL-1β, and IL-6 remained significant after adjusting for possible confounders.

Our findings are in agreement with other clinical and experimental studies. Ter Horst et al. investigated factors influencing individual cytokine responses in 534 healthy individuals of Caucasian origin. They found that monocyte-derived cytokine production (IL-1β, TNFα, IL-6, IFN-γ) after ex vivo blood stimulation with LPS or Candida albicans is higher in men (26). In another study, LPS-stimulated TNFα was nearly 30% lower in female than in male volunteers (27). In mice, LPS-challenged macrophages derived from males produce higher levels of pro-inflammatory cytokines than similarly treated female-derived cells (28). Constitutively higher cell surface expression of TLR-4 and its co-receptor CD14 on male macrophages could make these cells more sensitive to LPS exposure and result in observed sex-dependent differences in cytokine production (28).

A strength of our study is the multi-level validation of our findings through gene expression analysis. The RNA sequencing data revealed that the greater ex vivo synthesis of pro-inflammatory cytokines in males was mirrored by a significant up-regulation of their corresponding mRNA transcripts (TNFα, IL-1β, IL-6 and IL-12).

Since TLR-4 is a master regulator of immunity, there is a growing interest in developing TLR-4 antagonists or agonists to treat different diseases including stroke. ApTOLL, a DNA aptamer that inhibits TLR-4, is currently under investigation in patients with ischemic stroke (29). Preliminary studies showed that a dose of 0.2 mg/kg administered within 6 hours of stroke onset in combination with endovascular therapy is safe and associated with favourable outcomes compared with placebo. Our findings have potential implications for the development of immunomodulatory therapies targeting the TLR-4 pathway, which may exhibit sex-specific efficacy. Accordingly, future clinical trials should consider *a priori* stratification by sex, as the optimal therapeutic dose required to achieve comparable biological effects may differ substantially between men and women.

In previous reports, female patients tend to have worse functional outcomes after stroke than male patients (30, 31). The biological basis of sex differences in stroke outcomes remains unclear. Older age, more severe stroke, greater burden of pre-stroke comorbidities and more often pre-stroke dependency may account for worse outcomes in women, however, these factors do not fully explain sex differences in post-stroke outcomes (30). Experimental studies suggested that sex-specific differences in the immune response following stroke could be, at least partially, responsible for different outcomes between males and females (3). Our results, however, do not support this hypothesis. Previously, we reported that the reduced release of pro-inflammatory cytokines such as TNFα or IP-10 is associated with poor functional outcomes after stroke independently of age, stroke severity and pneumonia (15, 17). In the current study, despite significant differences in ex vivo cytokine synthesis, 3-month functional outcome did not differ between males and females. This may suggest that sex-related variations in ex vivo cytokine release are insufficient to produce a measurable effect on clinical outcome, or that the study was underpowered to detect an association between sex-specific immune responses and prognosis. Furthermore, we cannot exclude that selection bias may have influenced our results, as patients with pre-stroke disability and minor strokes were not included in the study.

We identified stroke severity, a monocyte count and the use of ARBs as predictors of ex vivo cytokine synthesis. Our findings are in line with previous studies. Emsley et al. found a negative correlation between NIHSS score on admission and ex vivo LPS-induced production of IL-1β, IL-6 and TNFα following acute ischemic stroke (32). Several studies on healthy volunteers showed that monocytes are a major source of cytokines in cultures of whole blood stimulated with LPS (24, 33). In turn, ARBs such as valsartan, candesartan or losartan inhibit *in vitro* production of pro-inflammatory cytokines by peripheral blood mononuclear cells (34).

Several limitations of our study should be acknowledged. First, cytokine production was assessed at a single time point only. Second, we were unable to account for infarct size and location, as well as women’s hormonal status (e.g., menopausal status or hormone replacement therapy), as potential confounders because these data were not collected. However, the women in our cohort had a mean age of 73.6 years, suggesting that the majority were likely postmenopausal, while the use of hormone replacement therapy in this population would be expected to be low. Furthermore, to minimize the risk of overfitting, our multivariable models were adjusted only for the most relevant predictors identified in the univariable analyses. Consequently, some factors previously reported to influence cytokine production (e.g., beta-blocker or statin use) were not included in the final models, as they were not significantly associated with cytokine production in our univariable analyses.

In conclusion, sexual dimorphism in the innate immune response following ischemic stroke, with males exhibiting higher ex vivo synthesis of pro-inflammatory cytokines, did not translate into sex-specific functional outcome.

## Data Availability

The datasets presented in this study can be found in online repositories. The names of the repository/repositories and accession number(s) can be found below: https://www.ncbi.nlm.nih.gov/sra/?term=PRJNA707400, PRJNA707400.

## Supplementary material

Supplementary Table 1 Results of univariable quantile regression with cytokine levels as dependent variables.

Supplementary Table 2 Results of multivariable quantile regression with cytokine levels as dependent variables.

Supplementary Table 3 Results of logistic regression with poor functional outcome at 3 months as a dependent variable.
