# Supplementary material for: Sex differences in ex vivo cytokine production and their association with outcome following ischemic stroke
Source: Front Immunol. 2026 Jul 28;17:1856274. doi: 10.3389/fimmu.2026.1856274 (PMC13457051; doi:10.3389/fimmu.2026.1856274)
Supplement: Supplementary file 1 [file Supplementaryfile1.docx]

Supplementary Material

Supplementary Table 1. Results of univariable quantile regression with cytokine levels as dependent variables

|  | TNFα | IL-12 | IL-10 | IL-6 | IL-1β | IL-8 | IP-10 |
| --- | --- | --- | --- | --- | --- | --- | --- |
| Age | -7.89 (-20.32; -2.68), P=0.19 | -0.08 (-0.13; -0.04), P=0.03 | 0 (-0.34; 0.29), P=1.00 | -42.44 (-95.08; 7.74), P=0.22 | -5.95 (-14,13; 1.77), P=0.25 | 3.72 (-7.88; 13.03), P=0.59 | -1.47 (-5.42; 2.26); P=0.55 |
| NIHSS score on admission | -24.60 (-43.05; 2.38), P=0.06 | -0.15 (-0.27; -0.06), P=0.03 | 1.11 (0.38; 1.52), P<0.01 | 91.63 (16.77; 240.45), P=0.22 | -11.17 (-23.24; 8.97), P=0.30 | 51.35 (22.91; 68.17), P<0.01 | -14.46 (-18.78; -10.07), P<0.01 |
| Male sex | 587.30 (366.10; 819.16), P<0.01 | 0.89 (-0.27; 1.86), P=0.17 | 6.98 (-4.73; 13.87), P=0.25 | 3179.34 (1163.76; 4573.28), P<0.01 | 511.54 (350.46; 704.56), P<0.01 | 194.92 (-149.68; 509.84), P=0.29 | 112.73 (24.03; 180.84), P=0.02 |
| Lymphocytes | 345.50 (137.55; 502.01), P=0.02 | 0.06 (-0.34; 0.77), P=0.91 | 1.10 (-0.58; 5.24), P=0.52 | 198.32 (-416.99; 1075.14), P=0.72 | 133.30 (15.74; 332.61), P=0.17 | -5.60 (-157.74; 223.86), P=0.96 | 52.04 (-2.49; 130.01), P=0.20 |
| Monocytes | 1322.62 (742.32; 1771.38), P<0.01 | -2.13 (-4.29; -0.78), P=0.05 | 40.35 (23.08; 50.50), P<0.01 | 10443.68 (7399.67; 12545.32), P<0.01 | 692.29 (347.42; 924.44), P<0.01 | 1882.33 (1548.06; 2837.08), P<0.01 | 60.75 (-78.73; 296.22), P=0.63 |
| Hypertension | 14.30 (-225.35; 418.90), P=0.94 | -0.09 (-1,73; 1.36), P=0.91 | 12.81 (3.16; 20.20), P=0.03 | 993.02 (-1034.57; 2359.7), P=0.32 | 228.81 (76.18; 412.51), P=0.08 | 527.75 (231.14; 779.96), P<0.01 | -5.93 (-131.84; 57.75), P=0.92 |
| Ischemic heart disease | 109.00 (-137.67; 494.09), P=0.61 | -0.32 (-1.68; 1.26), P=0.77 | 10.75 (2.93; 24.12), P=0.10 | 2761.39 (-143.70; 3341.56), P=0.02 | -23.34 (-447.79; 444.64), P=0.93 | 270.87 (-231.80; 949.55), P=0.43 | -13.12 (-122.79; 75.02), P=0.84 |
| Diabetes mellitus | 19.00 (-356.91; 242.89), P=0.91 | 0 (-3.64; 1.00), P=1.00 | -9.26 (-18.45; -0.93), P=0.12 | 308.55 (-1124.82; 1554.59), P=0.71 | -122.38 (-339.22; 250.93), P=0.48 | 3.13 (-276.67; 423.84), P=0.99 | 66.10 (-17.68; 158.51), P=0.23 |
| Atrial fibrillation | 102.50 (-363.58; 456.63), P=0.66 | -0.53 (-2.43; 0.94), P=0.48 | -7.73 (-13.96; 5.79), P=0.22 | -633.23 (-1972.19; 2663.49), P=0.65 | -63.71 (-323.29; 222.25), P=0.69 | 30.74 (-272.57; 497.47), P=0.89 | -45.72 (-141.99; 18.21), P=0.32 |
| Smoking | 240.70 (-89.41; 407.42), P=0.10 | 1.53 (0.30; 2.29), P=0.03 | 2.85 (-6.98; 11.15); P=0.58 | 1276.25 (-93.25; 3285.49), P=0.19 | 214.61 (-158.04; 454.13), P=0.17 | -57.10 (-433.53; 175.34), P=0.78 | 72.70 (-13.13; 141.47), P=0.13 |
| Reperfusion therapy | 70.00 (-398.62; 262.85), P=0.72 | 0.09 (-1.00; 1.40), P=0.87 | -7.49 (-14.95; 3.25), P=0.21 | 546.92 (-1238.94; 1538.80), P=0.55 | -340.45 (-558.47; -128.79), P=0.02 | -237.56 (-721.76; 52.66), P=0.38 | 0.02 (-84.49; 95.98), P=1.00 |
| Pre-stroke medication |  |  |  |  |  |  |  |
| Statins | 115.80 (-306.69; 484.26), P=0.56 | 0.22 (-0.61; 1.95), P=0.78 | 17.16 (7.54; 24.58); P<0.01 | 1755.88 (137.77; 3287.19), P=0.12 | 162.05 (-320.38; 579.80), P=0.59 | 276.59 (-54.86; 621.15), P=0.17 | 3.90 (-93.28; 72.88), P=0.94 |
| Acetylsalicylic acid | 28.30 (-328.56; 225.94), P=0.87 | -0.27 (-2.22; 1.25), P=0.75 | 3.36 (-8.98; 15.45), P=0.63 | 92.43 (-1311.90; 1909.96), P=0.93 | -221.99 (-433.14; 102.17), P=0.28 | 99.60 (-284.89; 522.93), P=0.70 | -16.44 (-104.64; 41.57), P=0.75 |
| β-blockers | 22.70 (-312.84; 322.47), P=0.90 | -0.63 (-1.89; 0.14), P=0.28 | 6.94 (-4.35; 20.50), P=0.32 | 1947.99 (-37.55; 3796.22), P=0.11 | 37.49 (-222.09; 309.93), P=0.81 | 550.09 (276.79; 871.85), P<0.01 | -6.98 (-89.17; 73.62), P=0.88 |
| α-blockers | 131.90 (-231.72; 977.86), P=0.70 | -0.49 (-4.20; 2.16), P=0.84 | 5.10 (-6.66; 18.39), P=0.51 | 92.43 (-1661.24; 3245.43), P=0.97 | -204.35 (-580.30; 297.61), P=0.43 | -44.78 (-592.55; 469.19), P=0.89 | 102.11 (-37.65; 232.48), P=0.36 |
| Diuretics | -332.60 (-532.86; 124.42), P=0.10 | -1.12 (-4.81; 0.17), P=0.19 | 2.32 (-7.55; 16.46), P=0.73 | -154.62 (-2163.93; 1826.04), P=0.90 | -206.35 (-413.53; 204.34), P=0.34 | 356.86 (-53.72; 595.94), P=0.07 | -157.10 (-267.98; -80.72), P<0.01 |
| ACE-Is | 35.20 (-270.34; 548.56), P=0.90 | 0.49 (-0.79; 1.72), P=0.36 | 4.61 (-8.59; 18.26), P=0.55 | 385.69 (-786.83; 2188.06), P=0.70 | 48.73 (-310.92; 334.71), P=0.81 | 241.67 (-199.30; 678.11), P=0.34 | 13.91 (-66.10; 106.77), P=0.77 |
| ARBs | -701.30 (-954.45; 151.87), P=0.07 | -4.19 (-10^6^; -2.02), P<0.01 | 2.32 (-8.46; 11.56), P=0.69 | -2404.55 (-3919.80; -301.41), P=0.03 | -343.33 (-601.23; -120.79), P=0.03 | -83.54 (-580.23; 192.60), P=0.77 | -133.07 (-212.55; -20.91), P=0.05 |
| Insulin | 257.00 (-202.49; 971.97), P=0.39 | -0.27 (-4.23; 3.61), P=0.89 | -0.66 (-16.13; 16.83), P=0.95 | 1284.26 (-677.48; 6303.82), P=0.52 | 563.29 (-231.35; 751.69), P=0.10 | 192.56 (-158.51; 907.66), P=0.58 | 111.98 (-77.89; 573.62), P=0.55 |
| Oral hypoglycemic drugs | -85.30 (-676.21; 214.55), P=0.75 | 0.31 (-2.17; 1.42), P=0.77 | -13.52 (-22.91; -6.84), P<0.01 | -541.39 (-2622.27; 721.59), P=0.62 | -143.70 (-449.15; 168.63), P=0.44 | 281.96 (-229.18; 596.01), P=0.29 | 56.22 (-42.22 – 158.64), P=0.43 |

Results are shown as β coefficients with 95% confidence intervals.

ACE-I: angiotensin-converting enzyme inhibitors; ARBs: angiotensin receptor blockers; NIHSS: National Institute of Health Stroke Scale

Supplementary Table 2. Results of multivariable quantile regression with cytokine levels as dependent variables

| Dependent variable | Independ variables included into a model |
| --- | --- |
| TNFα | Male sex: 407.28 (113.05; 668.47), P=0.02  NIHSS score: -34.90 (-57.56; -10.95), P=0.03  Monocytes: 1187.26 (773.68; 1742.63), P<0.01  Lymphocytes: 127.73 (-110.43; 333.156), P=0.39  ARBs: -124.18 (-625.87; 430.19), P=0.71 |
| IL-12 | Male sex: 0.43 (-0.85; 1.72), P=0.63  NIHSS score: -0.11 (-0.20; -0.005), P=0.10  Age: -0.07 (-0.11; -0.03), P=0.03  Monocytes: -2.33 (-3.90; -1.05), P=0.03  Smoking: 0.87 (-0.27; 1.75), P=0.34  ARBs: -1.94 (-3.13; -0.81), P=0.05 |
| IL-10 | Model without interaction terms  Male sex: -4.49 (-14.71; 7.08), P=0.47  Monocytes: 32.70 (18.92; 51.91), P<0.01  Hypertension: 9.12 (0.53; 14.83), P=0.07  Oral hypoglycemic drugs: -17.61 (-23.52; -8.30), P<0.01  NIHSS score: 0.04 (-0.39; 0.94), P=0.93  Statins: 7.95 (2.54; 13.62), P=0.12  Model with interaction terms  Male sex: 29.29 (5.48; 51.76), P=0.07  Monocytes: 75.18 (28.21; 99.34), P<0.01  Male sex x monocytes: -50.42 (-78.22; -18.36), P=0.04  Hypertension: 10.07 (2.24; 18.52), P=0.07  Oral hypoglycemic drugs: -18.44 (-24.95; -10.22), P<0.01  NIHSS score: -0.26 (-1.24; 0.95), P=0.71  Statins: 8.12 (1.67; 14.91), P=0.14 |
| IL-6 | Male sex: 1974.55 (5.58; 3064.27), P=0.04  Monocytes: 9769.51 (5436.05; 12651.86), P<0.01  Ischemic heart disease: 790.56 (-1328.76; 2464.70), P=0.56  ARBs: -1448.25 (-3131.96; 7.37), P=0.17 |
| IL-1β | Male sex: 310.07 (187.01; 514.00), P=0.01  Monocytes: 425.21 (53.12; 808.14), P=0.05  Reperfusion therapy: -292.68 (-452.18; -156.33), P=0.01  ARBs: -360.73 (-626.76; -172.83), P=0.04  Hypertension: 157.68 (26.08; 339.00), P=0.26 |
| IL-8 | Male sex: 157.53 (-173.95; 349.78), P=0.43  Monocytes: 1490.60 (1155.30; 2100.60), P<0.01  NIHSS score: 31.63 (1.92 – 49.45), P=0.07  Hypertension: 420.46 (-31.70; 682.66), P=0.08  Β-blockers: -15.06 (-351.00; 493.18), P=0.95  Diuretics: 104.70 (-204.83; 577.52), P=0.67 |
| IP-10 | Male sex: 36.87 (-73.74; 120.16), P=0.50  NIHSS score: -12.94 (-17.42; -7.88), P<0.01  Diuretics: -116.11 (-192.06; -63.54), P=0.04  ARBs: -18.80 (-127.91; 60.83), P=0.79 |

Results are shown as β coefficients with 95% confidence intervals. Multivariable models were adjusted for sex and for covariates associated with respective cytokine in univariable analyses (P < 0.10).

Supplementary Table 3. Results of logistic regression with poor functional outcome at 3 months as a dependent variable

|  | Univariable analysis | Multivariable analysis* | Multivariable analysis** |
| --- | --- | --- | --- |
| *Ex vivo cytokines* |  |  |  |
| TNFα ≤2256.4 pg/mL | OR: 2.52, 95%CI: 1.55 – 4.12, P<0.01 | OR: 4.41, 95%CI: 2.31 – 8.40, P<0.01 | OR: 4.59, 95%CI: 2.38 – 8.85, P<0.01 |
| IP-10 ≤346.5 pg/mL | OR: 2.95, 95%CI: 1.80 – 4.84, P<0.01 | OR: 3.05, 95%CI: 1.70 – 5.46, P<0.01 | OR: 3.14, 95%CI: 1.74 – 5.68, P<0.01 |
| IL-1β ≤1395.0 pg/mL | OR: 1.86, 95%CI: 1.14 – 3.01, P=0.01 | OR: 2.03, 95%CI: 1.15 – 3.59, P=0.01 | OR: 2.08, 95%CI: 1.17 – 3.71, P=0.01 |
| IL-6 >17231.8 pg/mL | OR: 1.50, 95%CI: 0.85 – 2.64, P=0.16 | OR: 0.86, 95%CI: 0.43 – 1.75, P=0.69 | OR: 0.86, 95%CI: 0.42 – 1.75, P=0.68 |
| IL-8 >2331.4 pg/mL | OR: 2.87, 95%CI: 1.70 – 4.84, P<0.01 | OR: 1.62, 95%CI: 0.88 – 2.98, P=0.12 | OR: 1.62, 95%CI: 0.88 – 2.99, P=0.12 |
| IL-10 >49.4 pg/mL | OR: 2.81, 95%CI: 1.71 – 4.62, P<0.01 | OR: 2.16, 95%CI: 1.21 – 3.85, P<0.01 | OR: 2.16, 95%CI: 1.21 – 3.85, P<0.01 |
| IL-12 ≤4.9 pg/mL | OR: 3.96, 95%CI: 2.36 – 6.63, P<0.01 | OR: 2.88, 95%CI: 1.62 – 5.12, P<0.01 | OR: 2.89, 95%CI: 1.63 – 5.14, P<0.01 |

*adjusted for age, NIHSS score, and monocyte count

** adjusted for age, NIHSS score, monocyte count, and sex (male: 0, female: 1)
